# A New Medical Journal

**Published:** 1878-03

**Authors:** 


### A NEW MEDICAL JOURNAL.

The first number of the "Detroit Medical News," was issued on the 21st of January. It is to be issued semimonthly, and this number contains twelve pages, all well filled with interesting and instructive matter on medical subjects.

Its projectors are impressed with the idea that they should have a more frequent vehicle of communication than heretofore, one that should take up new thoughts and issue before they have become stale.

This rapidity of movement may be well in some things, in others it is questionable.

We trust the "News" will prosper, live long and be happy.

